# Investigation of the effect of solubility increase at the main absorption site on bioavailability of BCS class II drug (risperidone) using liquisolid technique

**DOI:** 10.1080/10717544.2016.1250140

**Published:** 2017-02-06

**Authors:** Ahmed Khames

**Affiliations:** 1 Department of Pharmaceutics and Industrial Pharmacy, Beni-suef University, Beni-suef, Egypt and; 2 Department of Pharmaceutics and Pharmacy Technology, Taif University, Taif, Saudi Arabia

**Keywords:** Liquisolid, BCS class II drugs, risperidone, labrasol/labrafil mixture, bioavailability

## Abstract

BCS class II drugs usually suffer inadequate bioavailability as dissolution step is the absorption rate limiting step. In this work, the effect of solubility increase at the main absorption site for these drugs was investigated using risperidone as a drug model. Liquisolid technique was applied to prepare risperidone per-oral tablets of high dissolution rate at intestinal pH (6.8) using versatile nonionic surfactants of high solubilizing ability [Transcutol HP, Labrasol and Labrasol/Labrafil (1:1) mixture] as liquid vehicles at different drug concentrations (10–30%) and fixed (R). The prepared liquisolid tablets were fully evaluated and the dissolution rate at pH 6.8 was investigated. The formulae that showed significantly different release rate were selected and subjected to mathematical modeling using DE_25_, MDT and similarity factor (f2). Depending on mathematical modeling results, formula of higher dissolution rate was subjected to solid state characterization using differential scanning calorimetric (DSC), infrared spectroscopy (IR) and X-ray diffraction (XRD). Finally, the drug bioavailability was studied in comparison to conventional tablets in rabbits. Results showed that liquisolid tablet prepared using Labrasol/Labrafil (1:1) mixture as liquid vehicle containing 10% risperidone is a compatible formula with law drug crystallinity and higher dissolution rate (100% in 25 min). The drug bioavailability was significantly increased in comparison to the conventional tablets (1441.711 μg h/mL and 137.518 μg/mL in comparison to 321.011 μg h/mL and 38.673 μg/mL for AUC and Cp_max_, respectively). This led to the conclusion that liquisolid technique was efficiently improved drug solubility and solubility increase of BCS class II drugs at their main absorption site significantly increases their bioavailability.

## Introduction

In order to achieve a desired pharmacological response, the drug must reach a reasonable significant concentration in plasma which is mainly correlated to its solubility in GIT fluids (Vemula et al., 2010), except pinocytosis, all other drug absorption mechanisms from GIT require the presence of drug in solution form (Paradkar & Bakliwal, 2008).

For poorly water soluble drugs, mainly categorized as class II in BCS classification system (low solubility and high permeability), the dissolution is the rate determining step in the absorption process (Takano et al., 2008). So after oral administration, these drugs usually suffer slow and inadequate absorption from GIT, low bioavailability and probable mucosal damage (Savjani et al., 2012).

For oral drug delivery, improvement of drug solubility is the main challenge in drug formulations process (Khadka et al., 2014). Many approaches had been investigated and applied either alone or in combination for improving the solubility characteristics of poorly water soluble drugs including solvent change co-precipitation (Sertsou et al., 2002, Khanfar & Salem, 2010), solid dispersion (Hosny et al., 2013; Maruthapillai et al., 2015; Pereira et al., 2016), inclusion complexes with β-cyclodextrins (Gao et al., 2012; Sambasevam et al., 2013; Nair et al., 2014), nanosuspensions (Huang et al., 2013; Sahu & Das, 2014; Gora et al., 2016), microencapsulation (Aziz et al., 2012), soluble salts formation (Serajuddin, 2007), lyophilization (Dixit et al., 2011; Dixit & Kulkarni, 2012; Xu et al., 2016), solubilization (Rangel-Yagui et al., 2005; Seedher & Kanojia, 2008; Xu et al., 2012), and liquisolid technique (Khames, 2013; Kamble et al., 2014; Sanka et al., 2014; Badawy et al., 2016).

The liquisolid technique is a recent promising approach for improvement of drug water solubility in per oral drug delivery. In this technique, the insoluble drug is dissolved or suspended into suitable water miscible vehicle to prepare liquid medication. This liquid medication is simply mixed with selected powder additives of significant absorbing and/or adsorbing characteristics (carrier and coat) in calculated amounts according to the mathematical model described by Spireas (2002) to form dry, flowable powder mixture of good compressibility characteristics referred to as liquisolid compact (Burra et al., 2011).

This mathematical model depends on the flowable liquid retention potential (Ф-Value) of both carrier and coat materials at the selected carrier/coat ratio (*R*) to calculate the formula liquid load factor (Lf) which is directly used to calculated the amounts of carrier and coat materials that are usually enough to maintain good flow and compression properties to the prepared liquisolid compact (Spireas & Bolton, 1999). Being simple, economic, and easily applied on industrial scale, the liquisolid technique is highly suggested for larger pharmaceutical applications (Javadzadeh et al., 2007).

Other promising application of the liquisolid compact technique is improvement of photostability of liable drugs in solid dosage forms, where the photoprotective action of the selected excipients (carrier and coat) can be an alternative for the conventional tablet coat (Khames, 2013).

**Figure 1.**
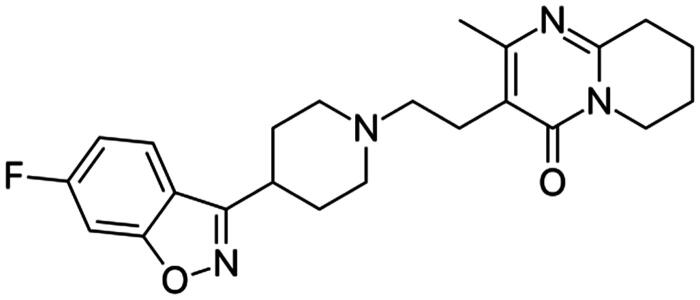
Risperidone chemical formula.

Risperidone is benzisoxazole derivatives of antipsychotic properties due to its high antagonistic effect on serotonin-5HT_2_ and dopamine-D_2_ receptors that magnifies its action in treatment of positive and negative schizophrenia with less extrapyramidal side effects and relapse probability (Rainer, 2008). Chemically it is C_23_H_27_FN_4_O_2_ (Bladania et al., 2008) (Figure 1). It is a weak base that is practically water insoluble, the solubility is pH dependent, it is highly soluble at acidic pH with significant decrease as pH increases up to pH 6.8 with minimum solubility at pH 8 (Saibi et al., 2012). Its bioavailability is about 70% with high protein binding ability (88%). It is extremely metabolized in liver to the active metabolite 9-hydroxyrisperidone (Novalbos et al., 2010).

Risperidone is categorized by biopharmaceutical classification system (BCS), as a class II drug of low solubility and high permeability, its absorption from GIT and bioavailability is mainly controlled by its dissolution rate (Silva et al., 2011). It is mainly absorbed (82.2% of the dose) in the upper part of the gastrointestinal tract, i.e. duodenum and jejunum of mild alkalinity, *in vitro*/*in vivo* correlation with level A (a point-to-point correlation) was proved in different dissolution media for Cp_max_ and AUC with low prediction error (<10%) (Saibi et al., 2012).

Based on the previously mentioned characteristics of risperidone, it was selected as a BCS class II drug model to investigate the effect of improvement of solubility at the main absorption site on its bioavailability. Risperidone was formulated as liquisolid tablets of high dissolution rate at intestinal pH (where the drug solubility declines with expected negative effect on its bioavailability) to increase the drug concentration at the absorption site in GIT and maximize the absorption process with consequent expected higher bioavailability.

## Materials and methods

### Materials

Risperidone and carbamazepine (kindly supplied from EIPICO, Egypt), Avicel PH 102 (FMC Corp., Philadelphia, PA), nanometer-sized amorphous silicon dioxide (SiO_2_) (Sigma-Aldrich, Steinheim, Germany), Transcutol HP, Labrasol and Labrafil (gift from Gattefosse SAS, Saint-Priest Cedex, France), Pharmaburst 500 (SPI Pharma, Inc., Wilmington, DE), Poly ethylene glycol 400, Tween 20 (Merck KGaA, Darmstadt, Germany), all other solvents are HPLC grade (Sigma-Aldrich, Steinheim, Germany).

### Methodology

#### Preparation of risperidone liquisolids

Several risperidone liquisolids were prepared using Avicel PH 102 and nanometersized amorphous silicon dioxide as carrier/coat mixtures with fixed ratio (*R*) equals 20 as the recommended ratio in the literature (Ahmed Khames & El Barry, 2006). The Φ-values of the carrier and the coating materials were calculated for different nonvolatile water miscible vehicles (namely, Transcutol HP, Labrasol, PEG 400, Tween 20 and Labrasol/Labrafil 1:1 mixture). The calculated Lf-value (the weight ratio of the liquid medication and carrier powder in the liquisolid formulations) was used for the determination of the amount of the carrier, while the coating material amount was calculated from the *R*-value. The calculated amounts of the carrier and coating materials supposed to maintain acceptable flow and compression properties for the prepared mixtures (Spireas, 2002).

In a porcelain mortar, the drug (4 mg) was manually mixed with each of the nonvolatile water miscible vehicles to prepare homogeneous mixtures of different drug concentrations ranging from 10 to 30%. To the prepared liquid solutions, Avicel PH 102 was added under continuous mixing followed by nanometer-sized amorphous silicon dioxide to adsorb the excess fluid. This mixing order was suggested to favor optimal release rate (Syed & Pavani, 2012). Finally, the prepared liquisolid mixtures were compressed on a suitable punch (single punch eccentric tablet press EP-1, Erweka, Germany) after addition of 10% of Pharmaburst 500 SPI (as disintegrant) and 1% magnesium stearate (as lubricant). The constituents for each formula are listed in (Table 1). A batch of 50 tablets for each liquisolid formula was prepared.

**Table 1.** Composition and pre-compression characterization of risperidone liquisolid formulae.

Formula	Vehicle type	LDC^a^	Lf	*Q*	*q*	Dis^b^	Unit dose weight (mg)	Angle of repose	Ci%	Hausner ratio
F1	Transcutol HP	10	0.243	165	8.25	17.325	192.48	32.22	17.84	1.21
F2		20		83	4.15	8.715	96.82	31.16	19.16	1.19
F3		30		55	2.75	5.775	64.16	31.62	18.18	1.18
F4	Labrasol	10	0.202	198	9.9	20.76	230.64	30.12	17.67	1.17
F5		20		99	4.95	10.395	115.49	29.86	16.16	1.19
F6		30		66	3.3	6.93	76.994	30.25	16.87	1.16
F7	PEG 400	10	0.104	385	19.25	40.425	449.12	31.12	20.01	1.12
F8		20		193	9.65	20.265	225.14	31.24	18.79	1.14
F9		30		128	6.4	13.44	149.32	32.11	18.89	1.17
F10	Tween 80	10	0.142	282	14.1	29.61	328.97	30.35	18.13	1.20
F11		20		141	7.05	14.805	164.43	31.63	18.87	1.18
F12		30		94	4.7	9.87	109.66	32.15	17.82	1.19
F13	Labrasol/Labrafil (1:1)	10	0.151	265	13.25	27.825	309.14	30.75	18.92	1.17
F14		20		133	6.65	13.965	155.15	31.41	19.37	1.17
F15		30		89	4.45	9.345	103.82	31.82	19.41	1.16

^a^Liquid drug concentration.

^b^ Disintegrant amount (mg).

#### Evaluation of the prepared liquisolid compacts

##### Pre-compression evaluation

The flow and compression properties of the prepared liquisolid mixtures were determined by measuring the angle of repose, compressibility index and Hausner ratio (Khames, 2013). Each recorded value is an average of three determinations.

##### Post-compression evaluation

Weight variation, hardness (TBH 325, Erweka, Germany), friability (TAR, Erweka, Germany), content uniformity (Kulkarni et al., 2012) and disintegration time (DST-3/6 automated disintegration tester, Logan, UT) quality control tests were applied onto 10 liquisolid tablets sample from each formula according to standardized pharmacopeial United State Pharmacopeia (USP) conditions (U.S. Pharmacopeia and National Formulary, 2002).

#### *In vitro* dissolution studies

The drug release rate from the prepared liquisolid formulations was investigated in the USP XXIV dissolution testing apparatus II (UDT-804 paddle dissolution apparatus, Logan, UT) in 900 ml of phosphate buffer (pH 6.8) as the dissolution medium maintained at 37 ± 0.5 °C at 50 rpm. At pre-determined time intervals (5, 10, 15, 25, 30, 45, 60 and 90 min) 5 ml sample was withdrawn with replacement. The absorbance of the drug was spectrophotometrically measured at *λ*_max_ 235 nm (Shimadzu UV/Vis double beam spectrophotometer) after filtration on 0.45 membrane filter. The cumulative percentage of drug release was calculated using an equation obtained from previously constructed standard calibration curve. For comparison, the dissolution rate of drug from conventional tablets prepared using the same excipients without adding the liquid vehicle by direct compression were also determined. The mean of six determinations was considered.

##### Statistical analysis of dissolution data

The dissolution data was statistically analyzed using post hoc one-way ANOVA test (Tukey mode) to declare the significance of the observed difference in dissolution profile of drug from the prepared liquisolid formulae in comparison to the prepared conventional tablet and plain drug at *p* value > 0.05 (IBM-SPSS, Inc., Chicago, IL)

#### Mathematical modeling of drug release

Depending on the results of statistical analysis, the liquisolid formulae that showed a significantly different dissolution profiles were compared through the calculation of the following parameters.

#### The mean dissolution time (MDT) (Bernal et al., 2014)

(1) $$\text{MDT}=\frac{\sum_{j=1}^{n}t_{j}^{\text{AV}}\times \text{Δ}Q_{j}}{\sum_{j=1}^{n}\text{Δ}Q_{j}}$$

where (*j*) is the sample number, *n* is the number of dissolution sample times, (*t_j_*^AV^) is the time at the midpoint between *t* and *t* −1 (calculated with (*t* + *t* − 1)/2), and (Δ*Q_j_*) is the additional amount of drug dissolved between *t* and *t* − 1.

#### Dissolution efficiency at 25 min (DE_25_) (Chella et al., 2014)

The dissolution efficiency (DE%) expresses the integrated area under the dissolution curve up to a certain time, *t*, as a percentage of rectangle area represents 100% dissolution in the same time.

(2) $$\mathrm{DE}=\frac{\int_{0}^{t}\mathrm{Qdt}}{Q_{100}\times t}\times 100$$

where (*Q*) is the percent of drug released as a function of time, (*t*) is the total time of drug release, and (*Q*_100_) is 100% drug release.

#### The similarity factor (f_2_) (Helmy et al., 2012)

(3) $$f_{2}=50\;\log\left\{{\left[1+\frac{1}{n}\sum_{t=1}^{n}W_{t}{\left(R_{t}-T_{t}\right)}^{2}\right]}^{-0.5}\times 100\right\}$$

where (*R_t_*) and (*T_t_*) are the cumulative percentage dissolved at each of the selected (*n*) time points of the reference and test product, respectively.

#### Optimization of the prepared liquisolid formulation

Depending on the results of the previous studies, risperidone liquisolid formula of the highest drug release rate was selected and subjected to further evaluation including the following.

#### Solid state characterizations and compatibility studies

The selected risperidone liquisolid formula, plain risperidone and physical mixture (without the liquid vehicle) were subjected the following studies.

##### Differential scanning calorimetric (DSC) studies

Samples (2–4 mg) were separately weighed into an aluminum pan of differential scanning calorimeter (Perkin-Elmer DSC4, Norwalk, CT), calibrated with purified indium standard (99.9%), and continuously purged together with a blank with nitrogen gas over a temperature range of (25–300 °C) at heating rate of 10 °C/min. The differential scanning calorimetric (DSC) thermograms were recorded and analyzed.

##### Infrared Spectroscopy (IR)

Samples (2–4 mg) were separately mixed with about 400 mg of dry potassium bromide powder and compressed into transparent disc under pressure of 10 000 to 15 000 pounds/inch^2^; and scanned in the range of 4000–500 cm^−1^ at ambient temperature using IR spectrophotometer (Shimadzu IR-435, Kyoto, Japan) their IR spectra were recorded and analyzed.

##### X-ray diffraction (XRD)

Samples were subject to X-ray diffraction (XRD) analysis on XRD-6000 X-ray powder diffractometer (Shimadzu, Japan) coupled with a standard Cu sealed X-ray tube with 40 kV voltage and 40 mA current. Data collection was performed at 2-theta of 5–60° in steps of 0.04 and scanning speed of 0.4 degrees per step. XRD charts were recorded and investigated for any change in the drug crystalline pattern.

#### *In vivo* evaluation of the prepared risperidone liquisolid tablets

Depending on the results of the previous *in vitro* evaluation studies, the selected liquisolid formula was subjected to *in vivo* evaluation in comparison to the prepared conventional risperidone (4 mg) oral tablets (compressed without liquid vehicle) in rabbits.

##### Animals

A group of 10 healthy male albino rabbits weighing 2.5–3.0 kg was used in this study. Rabbits were fasted overnight before drug administration with free access to water, and kept on the same diet during the study time.

##### Study design

A single dose bioavailability study was performed on two phases according to a randomized crossover design, so that each rabbit received the two treatments of selected liquisolid formula (Treatment a), and conventional (4 mg) oral tablet (Treatment b). A washout period of seven days separated between the phases. A control 2 ml sample of blood was withdrawn from each rabbit before drug administration.

The animal dose of drugs was calculated with reference to Paget and Barnes table (Laurence & Bacharach, 1964). Accurately weighed amounts of each treatment equivalent to the calculated animal dose was crushed and suspended in minimal volume of water and given orally to the animal by the aid of syringe.

##### Sample collection

At pre-specified time intervals (namely 0, 0.5, 1, 1.5, 2, 3, 4, 6, 8, 12, 24 and 48) hours post dose, 2 ml blood samples were collected from the cannulated ear vein in a small stoppered heparinized tubes and centrifuged at 3000 rpm for 10 min, the collected plasma samples were transferred by pipetting into pre-labeled polypropylene screw–cap tubes and immediately stored at −20 °C until analysis.

#### Determination of risperidone in plasma

Plasma samples were analyzed using a validated, sensitive, reproducible and accurate liquid chromatography/tandem mass spectrometry (LC-MS/MS) (Boonleang et al., 2010) briefly described as follow:

##### Chromatographic conditions

The isocratic mobile phase consisted of acetonitrile and 50 mM ammonium acetate pH 5.5 (35:65 v/v), delivered into the mass spectrometer’s electrospray ionization chamber at a flow rate of 0.3 ml/min. The ion spray voltage was set at 4000 V, drying gas flow rate 10 L/min, nebulizer pressure 45 psi, and drying gas temperature 325 °C. Quantitation was achieved by LC-MS/MS detection in positive ion mode using Carbamazepine as Internal standard on a QT mass detector.

##### Sample preparation for determination of risperidone

All frozen plasma samples were thawed at ambient temperature and prepared for work by solvent extraction method as follow: plasma sample (250 μl) and internal standard solution in methanol (10 μl) were vortexed in 10 ml glass tube, 0.1 N sodium hydroxide (75 μl) and 30% w/v aqueous sodium chloride solution (100 μL) were added and the mixture was vortexed for 1 min, ether (6 ml) was then added and sample was vortexed for other 2 min. The tube was then centrifuged for 5 min at 6000 rpm and the upper organic layer was transferred to a clean glass tube and evaporated to dryness at room temperature. Finally, the dried residue was dissolved in (75 μl) mobile phase and (10 μl) sample was injected into the apparatus using the autosampler. Risperidone concentration in unknown samples was calculated with reference to the data obtained from the constructed calibration curve in plasma.

##### Pharmacokinetics calculations

Plasma concentration-time curves were constructed and the pharmacokinetic parameters [namely: Cp_max_ (μg/mL), *T*_max_ (h), AUC_0–24_ and AUC_0–∞_ (μg h/mL), *K*_el_ (h^−1^) and *t*_1/2_ (h)] for each rabbit were calculated and manipulated using WinNonlin Professional 4.0.1 software (Pharsight Corp., Cary, NC).

#### Statistical analysis of pharmacokinetic data

The mean pharmacokinetic parameters was statistically analyzed using independent sample student *t*-test at *p* value > 0.01 and the confidence intervals were calculated (IBM-SPSS, Inc., Chicago, IL).

## Results and discussions

According to the formulae composition shown in (Table 1), 15 risperidone containing liquisolid formulae were prepared using different liquid nonvolatile water miscible vehicles at fixed carrier/coat ratios (*R *= 20), the drug solution in the prepared liquisolid mixture was varied from 10, 20 and 30%.

In this work, in addition to the commonly used solvents PEG 400 and Tween 20, versatile nonionic liquid surfactants (namely, Transcutol HP, Labrasol and Labrasol/Labrafil 1:1 mixture) were applied as nonvolatile water miscible vehicles to prepare different liquisolid formulations of higher drug release rate.

It was proven that the drug release rate from the liquisolid mixture is dependent mainly on the drug solubility in the applied water miscible liquid vehicle (Khanfar et al., 2013). So, selection of the previously mentioned nonionic surfactants as liquid vehicles was on the basis of their high solubilization power as described by their high HLB values (Gattefosse, 2010).

The prepared liquisolid mixtures were directly compressed into tablets without any additional processing steps, this gives the advantage of avoiding the wet granulation problems and save equipment.

In direct compression, to obtain a uniform product the powder mixture must uniformly flow into the tablet dies. Generally, the significance of flow in solid dosage form manufacture is related to its direct effect on different processing steps including weighing, mixing, filling and feeding through hopper to the die, so free flowing formula mixture is necessary to insure uniform weight content, accurate dosage and elegant product of high quality accepted by the market (Shanmugam, 2015).

Angle of repose, compressibility index, and Hausner ratio represent quantitative parametric indicators describe the powder flow. Angle of repose around 25° and Hausner ratio close to unity indicate good flow, while compressibility value greater than 20–21% refers to poor flow (Khames, 2013).

In this work, all prepared liquisolid mixtures can be considered of acceptable flow properties, where the angle of repose ranging from 29.86° to 32.22°, percentage of compressibility of maximum 20.01% and Hausner ratio values close to unity (Table 1). As the good flow is the large barricade and also a principal target in the liquisolid technique, these results indicate the accuracy of the calculated basic parameters including carrier and coat Φ-values for used nonvolatile water miscible vehicles and Lf-value at the selected carrier/coat ratio.

### Characterization of the prepared tablets

Characterization results presented in (Table 2) showed that the drug content was within the standardized pharmacopeial limit in all formulations (97.92–101.69%) with small acceptable weight variation. The prepared liquisolid formulae had acceptable mechanical properties and good breaking strength (46–68 N with maximum percentage weight loss of 0.77%) of noticeable correlation with tablet weight.

**Table 2.** Post-compression characterization of risperidone liquisolid formulation tablets.

Formula name	Average weight (mg) ± SD	Thickness (cm)	Diameter (cm)	Content uniformity (%) ± S.D	Friability (%)	Hardness (N)	Disintegration time (s)
F1	194.12 ± 0.661	0.15	0.6	98.39 ± 0.869	0.63	58	47
F2	94.97 ± 0.499	0.12	0.2	97.96 ± 0.991	0.69	55	42
F3	66.27 ± 0.211	0.11	0.2	100.75 ± 0.844	0.77	46	35
F4	232.31 ± 0.816	0.25	0.6	99.08 ± 0.261	0.67	56	42
F5	113.69 ± 1.039	0.13	0.2	97.89 ± 0.844	0.69	54	44
F6	77.11 ± 0.133	0.11	0.2	98.39 ± 0.587	0.72	49	40
F7	450.72 ± 0.731	0.22	0.8	98.99 ± 0.992	0.52	68	53
F8	223.21 ± 0.829	0.25	0.6	100.41 ± 1.340	0.64	58	45
F9	151.41 ± 0.654	0.13	0.6	101.69 ± 0.261	0.71	52	42
F10	329.17 ± 0.961	0.20	0.8	99.31 ± 0.844	0.59	64	52
F11	165.51 ± 1.071	0.13	0.6	100.71 ± 1.340	0.73	49	38
F12	112.16 ± 0.370	0.13	0.2	97.92 ± 0.869	0.71	49	40
F13	308.29 ± 1.129	0.18	0.8	99.28 ± 0.991	0.61	60	49
F14	156.33 ± 0.397	0.13	0.6	99.19 ± 0.519	0.69	56	44
F15	102.82 ± 0.157	0.12	0.2	98.72 ± 1.340	0.73	48	37

In this work, Pharmaburst 500 SPI (coprocessed blends of Mannitol, Starch, Crosspovidone, Crosscarmellose Sodium, Colloidal Silica and Silica) was applied as an efficient super-disintegrant of high drug compatibility, superior organoleptic properties, high solubility and very rapid disintegration due to combined wicking, swelling and elastic characteristics of crosscarmellose sodium and crosspovidone content (Nadavadekar & Koliyote, 2014). This could clearly explains the rapid disintegration of all prepared liquisolid tablets (53–35 s).

Being a BCS class II drug with level (A) *in vitro* release/*in vivo* absorption correlation (Saibi et al., 2012), poor solubility at alkaline pH (range from > 200 mg/ml at pH 2.1 down to 0.29 mg/ml at pH 7.6 and reaches 0.08 mg/ml at pH 8) (Anon), and more than 82% of the dose is absorbed from duodenum and jejunum, where the solubility significantly drops. The challenge here was mainly to improve risperidone solubility in simulated intestinal pH to insure higher drug concentrations at the main absorption site and increase drug bioavailability. For that, phosphate buffer (pH 6.8) was selected as discriminative dissolution medium to simulate intestinal dissolution conditions at the drug absorption site.

In previous work (Kaparthi & Babu, 2015), risperidone was formulated as liquisolid tablet formulae to investigate the effect of formula component (drug in liquid vehicle, binder and super disintegrant) concentrations and carrier level on drug release in 0.1 N HCl where the solubility of drug is already high. The prepared formulae were dependent on commonly used PG as liquid vehicle and the drug bioavailability from the prepared formulations were not studied.

To clarify any conflict with the previously mentioned published work, it is necessary to explain that this study depended on versatile nonionic surfactants of high solubilizing ability, high physiological safety and proven significant effect on absorption process as liquid vehicle (Transcutol HP, Labrasol and Labrafil) to prepare the liquisolid formulae and gave more concern to improve and test for risperidone (selected as BCS class II drug model) solubility at alkaline pH as simulated absorption site medium (where the drug shows minimum solubility). The drug bioavailability from the prepared liquisolid formulae was also studied.

### Dissolution data of the prepared liquisolids

**Figure 2.**
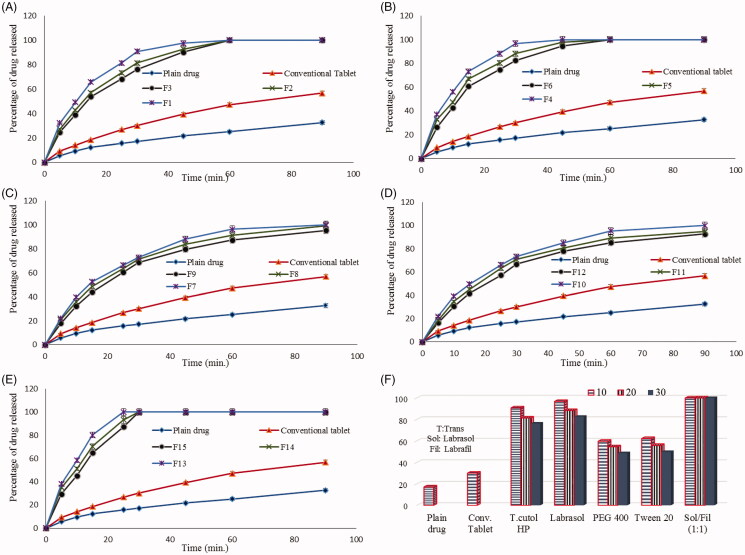
Dissolution data of risperidone from different prepared liquisolid tablet formulae in comparison to plain drug and conventional tablets.

Figure 2 shows the dissolution profiles of risperidone from different liquisolid tablet formulae in comparison to plain drug and conventional tablet in phosphate buffer (pH 6.8).

All prepared liquisolid tablet formulae showed higher drug release rates in comparison to risperidone powder and the conventional tablets up to 90 min, where percentage drug dissolved reached only 32.6% and 56.8% for plain powder and conventional tablets, respectively while this percentage exceeded 90% up to 100% from the prepared liquisolid tablets within the same time.

Previous studies (Khames, 2013) investigated the effect of the applied carrier/coat ratio (*R*) and coat type on the percentage drug release from liquisolid formulations. This work gave more attention to the effect of the applied liquid vehicle type and concentration on the drug release rate. For that, fixed (*R* = 20) was applied and a single coating material of hydrophilic nature (SiO_2_) to enhance tablet wettability was applied.

Further investigation to the dissolution data showed that, the drug release rate from liquisolid tablets varied with the applied liquid vehicle type and concentration. The percentage of drug released after 90 min from liquisolid formulae containing tween 20 (F10–F12) as liquid vehicle was 91.8, 96.7 and 100% while the percentage was 95.3, 99.3 and 100% from formulae containing PEG 400 (F7–F9) at drug concentration of 30, 20 and 10%, respectively (Figure 2C and D).

Liquisolid formulae containing Transcutol HP, Labrasol and Labrasol/Labrafil (1:1) mixture as liquid vehicles showed higher drug release rate, where the percentage of drug released reached 100% within 60 min at all drug concentrations and this could be related to the high solubilizing power of these vehicles.

Transcutol HP was recommended as a safe and physiologically compatible co-solvent to improve risperidone solubility, where the drug showed high solubility in plain Transcutol (5.163 × 10^−3^ in comparison to 1.1 × 10^−7^ in pure water) and most of its water co-solvents (Shakeel et al., 2014).

On the other hand, Labrasol is extensively used as a powerful surfactant for increasing the solubility and bioavailability of low-solubility drugs (Strickley, 2004), Labrasol 15% (w/v) in water showed 50 fold increase in solubility of poorly water soluble drug piroxicam (Karataş et al., 2005).

This could clearly explains the high increase in drug dissolution rate from theses formula and also describes the effect of vehicle concentration, where the percentage of drug released from Transcutol HP containing liquisolid formulae (F1–F3) was 90.3, 92.8 and 97.6% in comparison to 94.6, 97.7 and 100% from Labrasol containing formula (F4–F6) in only 45 min at drug concentration of 30, 20 and 10%, respectively (Figure 2A and B).

Labrasol based formulations usually suffer drug precipitation on dilution within aqueous media, this precipitation process decreases the amount of drug available for absorption which is a principal cause of decreasing the oral bioavailability especially for poorly water-soluble drugs. Mixtures of Labrasol with other pharmaceutical additives including lipids, surfactants and cosolvents significantly inhibit and/or retard this precipitation process on dilution (Dai et al., 2011).

So in this work Labrasol/Labrafil (1:1) mixture was applied as liquid vehicle of high a powerful solubilizing power and higher drug tolerating ability.

Results in (Figure 2E) showed that the drug was completely dissolved in 30 min and the percentage of drug released was 87.1, 93.3 and 100% from Labrasol/Labrafil (1:1) containing formulae (F13–F15) as liquid vehicle after only 25 min at drug concentration of 30, 20 and 10%, respectively.

The histogram illustrated in (Figure 2F) shows the percentage of drug release from different prepared liquisolid tablets in comparison to plain drug and conventional tablets after only 30 min. The figure clearly summarizes the dissolution results and shows the effect of liquid vehicle type and concentration on drug release rate, the drug release rate increased as the vehicle amount increased and also was dependent on vehicle solubilizing power where it reached 100% from Labrasol/Labrafil (1:1) liquisolid based formula regardless of the applied ratio.

### Statistical analysis of dissolution data

**Figure 3.**
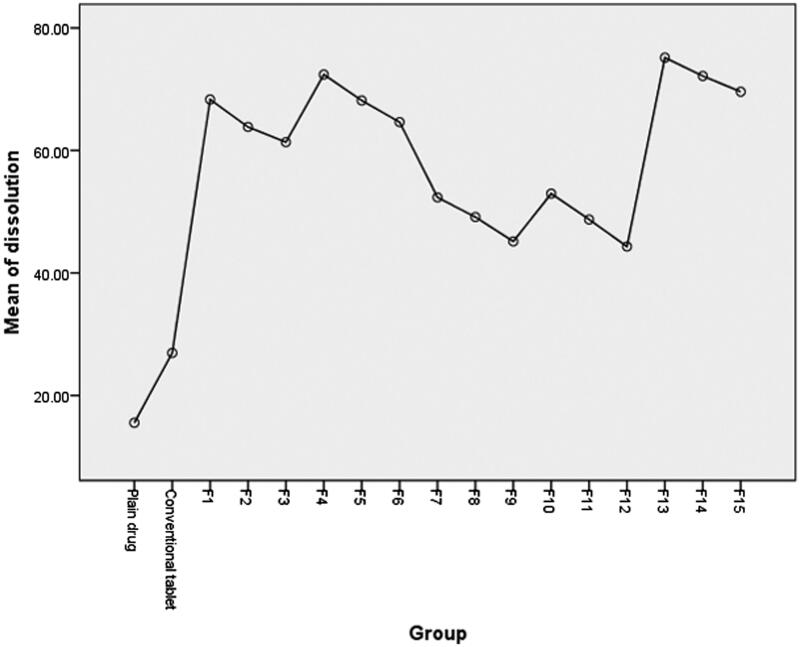
Graphical presentation of post hoc statistical analysis of dissolution data.

Post hoc one-way ANOVA test was applied to statistically analyze the dissolution data (more than two groups are compared). Results presented in (Table 3) indicated a significant difference between the dissolution profiles of the drug at the selected probability level (*p* > 0.05). Further data interpretation according to post hoc (Tukey mode) analysis, (Figure 3), which is mainly applied to precisely detect which mean of significantly different group of means (more than two) is different from others (Armstrong et al., 2000), showed that only dissolution profiles of drug from liquisolid tablet formulae F4, F13 and F14 are significantly different from other compared groups including plain drug and conventional tablet.

**Table 3.** One way ANOVA test of dissolution data.

	Sum of squares	df	Mean square	*F*	Sig.
Between groups	39 658.936	16	2478.684	2.261	0.006
Within groups	149 081.813	136	1096.190		
Total	188 740.749	152			

### Mathematical modeling of the dissolution data of the selected liquisolid formulae

Results showed that, the DE 25 increased to reach 100, 98.29 and 97.92% for F13, F14 and F4 in comparison to 19.7 and 31.28% for plain drug and conventional tablets, respectively. The MDT also significantly shortened to be 9.17, 10.88 and 11.51 min for F13, F14 and F4 in comparison to 32.49 and 33.59 min for plain drug and conventional tablets, respectively

For a certain dosage form, it was agreed that the dissolution profile is more precise and better descriptive for product performance than a single point dissolution test (PAL et al., 2014). For comparison, the selected parameter should expresses the degree of similarity between two profiles on the basis of high sensitivity to major differences at any time point. For that, Moore and Flanner suggested an independent mathematical efficient comparative parameter, that inversely proportional to the average squared difference between the two profiles, to measure how close they are entitled similarity factor (f2). FDA standardized for f2 value ≥ 50 indicates similarity between two dissolution profiles and f2 = 100 refers identical profiles (Ocaña et al., 2009).

Hence f2 values are only considerable when both products release ≥ 85% of labeled dose (Ocaña et al., 2009), only similarity factor for F4, F13 and F14 were compared. Results indicated that the three compared liquisolid formulae are identical with higher similarity between F4 and F14 (f2 = 74.6) than F4 and F14 when compared to F13 as a reference (f2 = 64.68 and 64.44), respectively.

Depending on the previous evaluation results, F13 prepared using Labrasol/Labrafil (1:1) mixture as liquid vehicle containing 10% risperidone was considered the best formula and selected for further compactability and *in vivo* evaluation studies.

### Solid-state characterization

**Figure 4.**
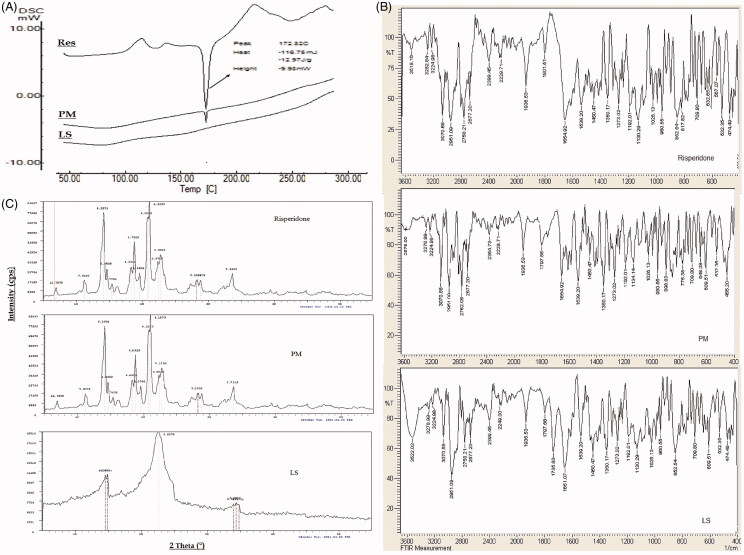
Solid state characterization of risperidone liquisolid formula (F13) using A) DSC, B) IR and C) XRD.

Results of solid-state characterization of the selected liquisolid tablet formula (F13) are summarized in Figure 4.

The DSC thermogram of risperidone showed a sharp endothermic melting peak at 172.82 °C indicting purity of the used drug sample. In physical mixture sample, this peak retained in the same position with lesser intensity (mainly due to drug dilution within the mixture) indicating compatibility of the mixture. The DSC thermogram of the selected liquisolid formula mixture (F13) showed a complete disappearance of the drug melting peak and decrease in melting enthalpy to be an evidence of loss of drug crystallinity mainly due to dissolution within the prepared formula mixture in an amorphous form, the absence of any additional peaks within the thermogram insures absence of any interaction and chemical compatibility within the liquisolid formula mixture (Figure 4A).

Results of IR studies (Figure 4B) indicated that, the main characteristic functional groups of risperidone namely CH3 stretching (2958 cm^−1^), CF stretching (1350 cm^−1^), C = O stretching (1654 cm^−1^), C = N stretching (1600 cm^−1^), aromatic CH stretching (3070 cm^−1^) and C = N–O stretching of 1,2 benzisoxazole (1539–1620 cm^−1^) are all appeared in the IR spectrum of plain drug and retained in the spectra of both physical mixture and the selected liquisolid formula eliminating the possibility of any chemical interaction and insures the compatibility of the selected excipients.

XRD is an efficient and valuable tool for identifying the crystallinity nature of materials and also a suitable, trusted tool for comparison. Figure 4C shows the XRD pattern of risperidone in comparison to physical mixture and the selected liquisolid formula mixture. The XRD pattern of plain drug exhibited sharp, intense and less diffused peaks at 2 theta angels of 6.9, 11.19, 14.08, 14.58, 14.60, 14.34, 18.27, 18.73, 19.53, 20.73, 20.99, 22.37, 22.76, 28.22, 28.80 and 33.58° indicating the high crystalline nature of drug. In the XRD patterns of physical mixture, the drug characteristic peaks were almost retained at their original diffraction angles with similar intensity indicating drug retained its crystallinity nature within the mixture. The XRD pattern of optimized liquisolid formula showed major change including peak disappearance and decreased intensities where only very weak peaks were recorded at 2 theta angles of 14.54, 14.82, 22.62, 33.99, 34.36 and 34.8° with attenuated diffraction. These changes on the drug characteristics peaks at same diffraction angles ascertain the solid-state transformation of risperidone to amorphous form within liquisolid formula. These results are in accordance with that recorded by DSC studies.

### *In vivo* characterization

**Figure 5.**
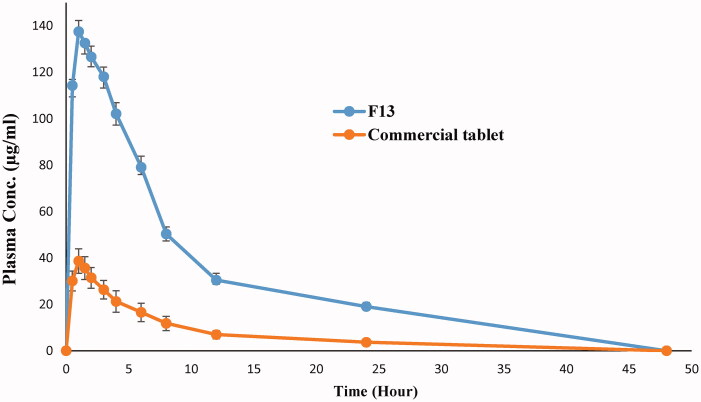
Mean plasma concentration time curve of risperidone liquisolid formula (F13) and conventional tablet in rabbits.

Figure 5 shows the mean plasma concentration time curve of drug from liquisolid formula F13 in comparison to conventional tablets while the main characteristic pharmacokinetic parameters are presented in (Table 4).

**Table 4.** Mean pharmacokinetic parameters of risperidone liquisolid formula (F13) and conventional tablet in rabbits.

Parameter	F13	Commercial tablet
Cp_max_	137.518 ± 23.2	38.673 ± 4.61
*T*_max_	1 ± 0.215	1 ± 0.199
AUC_0–48_	1213.111 ± 85.39	276.984 ± 14.72
AUC_0–∞_	1441.711 ± 93.88	321.011 ± 19.81
*K*	0.0619 ± 0.0192	0.10545 ± 0.0843
T1/2	7.556 ± 0.421	6.619 ± 0.311

The extent of drug absorption from GIT is expressed by Cp_max_ and AUC while *T*_max_ mainly signifies absorption rate (Hauschke et al., 2007). According to the collected pharmacokinetic parameters, the prepared liquisolid formulae showed higher extent of drug absorption (1441.711 μg h/mL and 137.518 μg/mL in comparison to 321.011 μg h/mL and 38.673 μg/mL for AUC and Cp_max_, respectively) without any considerable change in drug absorption rate where *T*_max_ kept constant at 1 h. These results could be correlated to the higher solubility of the drug from prepared liquisolid formula especially at the main absorption site in GIT (duodenum and jejunum) where increased drug wettability and degree of subdivision due to Labrasol/Labrafil mixture allowed exposure of more drug to the absorption site. The results could be discussed according to Fick’s first law of diffusion.

(4) J=-D(dc/dx)

Where (*J*) is the flux (absorption rate), (*C*) is the drug concentration at absorption membrane, (*X*) is the membrane length and (*D*) is the diffusion coefficient.

Where, the diffusion rate through the duodenum and jejunum membrane (absorption rate) increases as the drug concentration at the absorption site increase.

These results declare that the solubility increase for BCS class II drugs at the absorption site is valuable and of major significance to overcome absorption problems and increase their bioavailability.

***Statistical analysis of data*** indicated a significant difference between the calculated pharmacokinetic parameters from both conventional tablet and selected liquisolid formula (F13) with *p* value of <0.001, confidence interval are listed in (Table 5). The 95% confidence interval describes a range of mean values with 95% certainty that contains the true population mean, in other words it is 95% chance that the repetition of the experiment with the same conditions will obtain the calculated mean within the calculated range. According to the study results it could be noted that, the calculated 95% confidence intervals of the calculated pharmacokinetic parameters not contain zero value assuring significance of the detected difference and supporting rejection of null hypothesis, also the narrow range of the calculated confidence intervals indicates precision and strength of the deduced results.

**Table 5.** Confidence intervals of the statistically calculated mean differences of risperidone pharmacokinetic parameters.

Parameter	*p* value	Mean difference	Confidence interval
Cp_max_	<0.001	98.845	94.074–103.616
*T*_max_		0.9364	0.504–1.369
AUC_0–48_		936.127	880.497–991.757
AUC_0–∞_		1120.966	1049.192–1192.206
*K*		−0.0136	− (0.0201–0.007)

## Conclusions

Liquisolid technique is simple and efficient method to prepare a solid dosage form of high drug solubility with good pre and post characterization properties. Labrasol/Labrafil (1:1) mixture is optimum vehicle to prepare compatible risperidone liquisolid tablets of high dissolution rate at the main absorption site (pH 6.8). The prepared Labrasol/Labrafil (1:1) based liquisolid formula at 10% drug concentration showed rapid dissolution at targeted pH, where complete drug release (100%) occurred only within 25 min. The drug bioavailability was significantly increased as indicated by Cpmax and AUC values. All these data led to the final conclusion that solubility increase of BCS class II drugs at their main absorption site significantly increases their bioavailability.
